# Cost-Effectiveness Analysis of a Heart Failure Management System in the United States

**DOI:** 10.36469/001c.130066

**Published:** 2025-03-17

**Authors:** Antonia Bosworth Smith, Ubong Silas, Alex Veloz, Peter Mallow, Barbara Pisani, Diana Bonderman, Rhodri Saunders

**Affiliations:** 1 Coreva Scientific GmbH & Co. KG, Königswinter, Germany; 2 ZOLL Medical Corporation, Pittsburgh, Pennsylvania, USA; 3 Xavier University, Cincinnati, Ohio, USA; 4 Atrium Health Wake Forst Baptist, Winston-Salem, North Carolina, USA; 5 Division of Cardiology and Emergency Medicine Favoriten Clinic, Vienna, Austria

**Keywords:** remote monitoring, wearable monitor, cardiac failure, heart decompensation

## Abstract

**Background:** The disease burden of heart failure is mainly driven by high hospital readmission rates. Remote monitoring devices can be used to assess the status of patients after discharge and identify early signs of worsening symptoms. Initial studies indicated that Heart Failure Management System (HFMS), a novel monitoring device, can prevent hospital readmission. **Objective:** To determine the cost effectiveness of HFMS compared with standard of care (SOC) in the United States. **Methods:** A Markov model was developed to follow patients after their discharge from index hospitalization for heart failure. The costs and outcomes were estimated for 5 years. The patient cohort was initially in “outpatient care,” where they are at risk of an emergency room visit or hospital readmission. If hospitalized, patients returned to a second outpatient care health state. An “escalation of care” (eg, surgical intervention) may have removed patients from the intervention. The model took the payer perspective with costs in 2022 US dollars. The incremental cost-effectiveness ratio measured effectiveness through hospital readmissions. The willingness-to-pay threshold was set to the published cost of a heart failure rehospitalization ($10 737). Sensitivity and scenario analyses explored the robustness of the model to changes in inputs. **Results:** Compared with SOC, HFMS reduced the mean cost of care by 6723(155 122 vs $161 846) over the 5-year period. The mean number of hospital readmissions was reduced to 1.075 with HFMS from 1.201 with SOC (-0.126 events). The incremental cost-effectiveness ratio showed that HFMS was a dominant strategy compared with SOC, leading to reduced costs and hospital readmissions in 93.4% of the 1000 Monte Carlo simulations; 94.1% of the simulations fell below the willingness-topay threshold. Savings with HFMS emerged from the third month. **Discussion:** The results indicated the cost-effectiveness of HFMS compared with SOC. The sensitivity analyses supported this finding. Reducing costly hospital readmissions may help to alleviate the burden of heart failure. Longer-term data on HFMS are encouraged to confirm or contest the model outcomes. **Conclusions:** The use of HFMS is expected to save costs and reduce hospitalizations over a 5-year period compared with the current SOC.

## BACKGROUND

Heart failure is a substantial burden on the US healthcare system. The American Heart Association (AHA) calculated that an adult receiving care for heart failure had an average healthcare expenditure of $18 000 per annum more than an individual of the same age, sex, and comorbidity burden.^1^ A large part of the cost burden is in direct healthcare costs,^1^ and a review of heart failure literature showed that 90-day hospital readmission rates are between 16.7% and 31.2%, with each hospital readmission costing on average between $10 737 and $17 830.^2^

Containing healthcare costs is of increasing concern as health systems struggle with inflation, staff shortages, and other financial constraints. Heart failure is particularly expensive, with the majority of the costs occurring during inpatient care.^3^ Since 2010, the Hospital Readmissions Reduction Program has offered incentives to reduce costly, unnecessary hospital visits.^3^ Without optimized patient management, this burden will only increase, as the prevalence of heart failure is predicted to continue rising from its current 6.7 million people in the United States.^4^

Guideline-directed medical therapy is the mainstay of heart failure management and prescribed for all patients who will tolerate it, along with lifestyle changes.^5^ The management of certain cases may also include surgical or catheter-based intervention.^6^ Still, heart failure patients remain at high risk for hospitalization. Monitoring is needed to allow for timely identification of worsening symptoms.^7^ The current standard of care (SOC) for monitoring of heart failure patients involves routine follow-up (with or without blood pressure or body weight monitoring); this approach relies on the self-assessment of the patient.^5^ Many factors, such as the complexity of monitoring and health literacy, likely impact the quality of current self-monitoring. A review of patient monitoring in heart failure has noted that successful strategies “minimize patient-driven data collection” and promote “passive strategies for data collection and transmission.^8^”

The potential for remote patient monitoring to improve heart failure care was emphasized by the 2022 AHA/ACC/HFSA Guideline for the Management of Heart Failure.^9^ Unlike traditional approaches that rely heavily on patients reporting their own symptoms or weight changes, which are inherently subjective and often delayed, there is a need for real-time, objective, quantifiable insights into critical physiological parameters.^9^ The *Journal of the American College of Cardiology* (JACC) released a scientific statement supporting remote patient monitoring for heart failure that is coupled to a system of care.^10^ Monitoring alone only provides data but does not prevent events. The JACC statement encourages acting on data provided by remote monitoring, in essence, remote management, in the hope that remote management could help control symptoms and avoid hospitalizations. Remote monitoring of physiological parameters is becoming increasingly common in many disease areas. Through either implantable monitors or wearable devices, remote heart failure monitoring and management has been shown to allow for early detection of worsened symptoms.^11,12^ Monitoring may reduce the unneeded readmissions and allow physicians to safely discharge patients sooner from the hospital.^13^

In addition, the data from monitoring devices could be used to inform appropriate changes in the management of the patient.^13^ The scientific statement from JACC highlighted that the largest decrease in hospitalizations was found when congestion with cardiac filling pressures or lung water content was tracked.^10^ The HFMS collects multiple physiological measures and uses a radiofrequency sensor to estimate thoracic fluid content. This noninvasive, wearable monitoring system allows for continuous monitoring of patients by an independent diagnostic testing facility.^14^ The treating physician is alerted when potentially actionable events occur and has been shown to reduce heart failure readmissions, emergency room (ER) visits, and deaths within 90 days of discharge.^14^ The aim of this study was to assess the cost-effectiveness of HFMS compared with SOC in the United States.

## METHODS

A Markov model was used to represent a hypothetical cohort of patients in the United States who have recently been diagnosed with heart failure. The model was designed in accordance with good practice guidelines^15^ and was reported according to the Consolidated Health Economic Evaluation Reporting Standards (CHEERS 2022) checklist (see **Online Supplementary Material**).

The primary model output was the incremental cost-effectiveness ratio (ICER), defined as the additional cost per heart failure hospital readmission avoided. The model estimates the mean total cost per patient and the mean number of hospital readmissions per patient. With these two values for both SOC and HFMS, the ICER was calculated as:

$$ICER=\frac{CostsofHFMS-Costofstandardofcare}{EffectivenessofHFMS-Effectivenessofstandardofcare}$$

**Figure 1.**
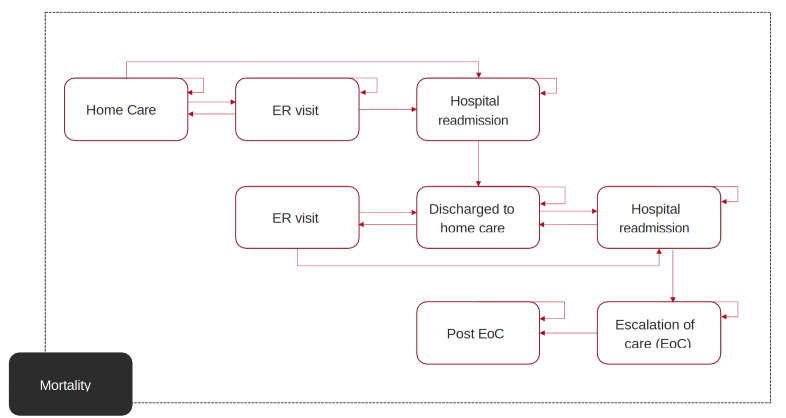
Markov Model Structure Adapted from Schmier et al^16^ Abbreviations: EoC, escalation of care; ER, emergency room.

The model structure was adapted from a previously published model on heart failure monitoring and developed using Excel (Microsoft Inc.).^16^ The Markov model was designed with 9 health states as illustrated in **Figure 1**. Two cohorts were modeled, one for the SOC group and one for the intervention group. The hypothetical population reflects the heart failure population from the BMAD study (Benefit of Microcor in Acute Decompensated Heart Failure with RF-Directed Therapy).^14^ This includes all heart failure patients who have recently been discharged for an acute decompensation event, regardless of New York Heart Association class or ejection fraction classification. The data from the intervention group was informed by the treatment arm of the BMAD trial for the HFMS device.^14^ The model examined a 5-year time horizon from the perspective of an insurance payer. A cycle length of 1 day was used. The willingness-to-pay (WTP) threshold was set at $10 737 per avoided rehospitalization, as informed by Osenenko et al.^2^

All patients enter the model in the “outpatient care” state. The dotted line indicates that movement to death is possible from all health states.

The patient cohort enters the model through the initial state of “outpatient care,” following discharge for an index heart failure hospitalization. The patient cohort could remain in this state, or they could be admitted to the ER (ER visit) or directly to the hospital for a hospital readmission. Patients in the “ER visit” state could also be readmitted after their ER visit, that is move directly to “hospital readmission,” or be discharged back home (“outpatient care”). After hospital readmission, the cohort is moved to the “discharged to outpatient care” state, thus allowing for the risk of subsequent readmissions and ER visits to be accounted for.

If any part of the cohort experiences a subsequent readmission, they are at risk of requiring an “escalation of care” (eg, a surgical or catheter-based intervention). If a patient receives an “escalation of care” then they are discharged to “post EoC,” where they remain until the end of the time horizon or death. Death is possible from any state in the model. An annual discount rate of 3% was applied to all cost outcomes after the first year.

The model inputs (**Table 1**) were sourced from a previously published systematic literature review (PROSPERO registration number CRD42023410084). Additional hand searches were used to supplement the cost data. The clinical data on HFMS was taken from the BMAD trial result and ZOLL Medical data on file.^14^

**Table 1.** Base Case Model Inputs

**Parameter**	**Value (CI)**	**Source**
Event rates		
HFMS impact on hospitalization, hazard ratio	0.62	Boehmer et al^14^
HFMS impact on mortality, risk ratio (95% CI)	0.66 (0.24; 1.82)	ZOLL data on file, additional analysis of data presented in Boehmer et al^14^
Hospitalization rate **at** 3 months, SOC (%) (95% CI)	32.55 (24.03; 41.63)	Silas et al^a^
Hospitalization rate **after** 3 mo: SOC (%) (95% CI)	52.21 (39.28; 64.99)	Silas et al^a^
Emergency room visit rate **at** 3 mo: SOC (%) (95% CI)	13.83 (8.21; 20.49)	Silas et al^a^
Emergency room visit rate **after** 3 mo: SOC (%) (95% CI)	50.0 (29.93; 70.07)	Silas et al^a^
Mortality rate **at** 3 mo: SOC (%) (95% CI)	3.46 (2.12; 5.06)	Silas et al^a^
Mortality rate **after** 3 mo: SOC (%) (95% CI)	9.13 (6.49; 12.14)	Silas et al^a^
Readmitted patients requiring EoC (%)	2.50	Assumption
Resource use		
Cost of HFMS per month ($)	1300	Assumption
Wear time of HFMS	2	Boehmer et al^14*^
Outpatient care costs per day ($) (assumed variance)	50 (35; 65)*	Obi et al^17^
Emergency room cost per visit ($) (lower and upper assumed 95% CI)	997 (482; 1458)	White-Williams et al^18^; Blum and Gottlieb^19^
Cost of hospital stay per day ($) (lower and upper assumed 95% CI)	2665 (2572; 4828)	Olchanski et al^20^
Intensive care unit stay per day ($) (assumed variance)	3417 (2392; 4442)b	Gershengorn et al^21^
Outpatient care costs after readmission ($) (assumed variance)	236 (212; 259)	Fitch et al^22^

Abbreviations: CI, confidence interval; HFMS, Heart Failure Management System; SOC, standard of care.

^a^Silas U, Hafermann J, Bosworth Smith A, Veloz A, Saunders R, Steidley D. Clinical outcomes in heart failure monitoring: a pooled rates analysis. *Am J Manag Care*. 2025;31(7).

^b^Variance was assumed to be 30% for costs when not reported. All costs were inflated to 2022 costs were necessary.

## Model Assumptions

The following assumptions were made to estimate the cost effectiveness over the 5-year time horizon:

- After 90 days the rate for hospital readmissions, mortality, and ER visits were assumed to be the same for HFMS (intervention arm in the BMAD trial) and SOC. This was the more conservative approach until longer-term outcome data are available.
- The rates of events in years 2, 3, 4, and 5 were taken to be the same as those in year 1. The assumption was made due to the lack of long-term data.
- A fixed percentage of heart failure patients (2.50%) who had repeated hospitalizations were assumed to have EoC.

Probabilistic and one-way sensitivity analyses accounted for uncertainties in the model inputs. The one-way sensitivity analysis, presented as a tornado plot, identified the model inputs that have the largest impact on the results. In this analysis, each input was individually increased and decreased by 10%. The results of the probabilistic sensitivity analysis are presented as a 95% credible interval (Crl) and the ICERs were plotted on the cost-effectiveness plane. This is created by simultaneously varying the input based on the attributed variance for each input. The distributions for each input were normal, log-normal, gamma, or beta distribution for continuous, relative risk, cost, and probability inputs, respectively. If not given by or imputed based on the literature, a variance of 10% or 30% was assumed for clinical and cost inputs, respectively. The probabilistic sensitivity analysis addresses the uncertainty in the model inputs, and by repeating it multiple times, a large range of potentially true inputs are created. In this model, 1000 iterations of the model were done.

## RESULTS

The mean cost for 1 heart failure patient for 5 years was $155 122 for HFMS and $161 846 for SOC (**Table 2**), hence demonstrating a cost reduction of $6723 at 5 years with the use of heart failure monitoring. The HFMS cohort experienced fewer hospital readmissions (improved effectiveness) than the SOC (mean, 1.075 vs 1.201 per patient). With a reduction in care costs and fewer hospital readmissions, HFMS dominated SOC in the base case analysis and no ICER was calculated.

**Table 2.** Model Base Case Results per Patient

	**Cost**	**Incremental Costs**	**Effectiveness**	**Incremental Effectiveness**	**ICER**
SOC	161 846		1.201		
HFMS	155 122	-6723	1.075	-0.126	Dominant

Abbreviations: HFMS, Heart Failure Management System; ICER, incremental cost-effectiveness ratio; SOC, standard of care.

**Figure 2.**
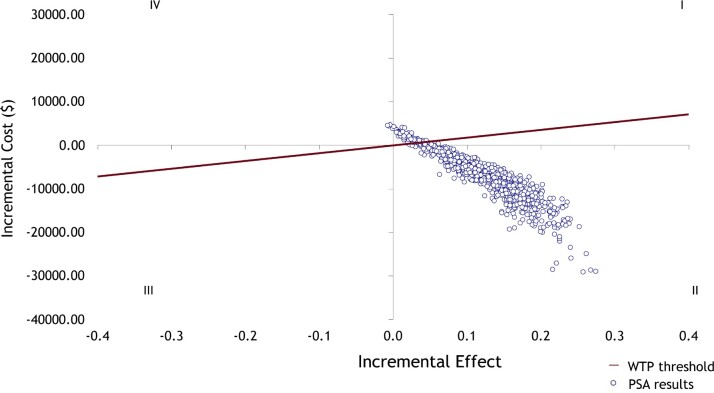
Cost-effectiveness Plane Showing the Scatterplot of 1000 Monte Carlo Simulations Abbreviations: PSA, probabilistic sensitivity analysis; WTP, willingness to pay.

The probabilistic sensitivity analysis (**Figure 2**) found that 93.4% of the results were in quadrant II (dominant), with HFMS remaining cost saving and more effective. 0.4% of the simulations resulted in SOC dominating HFMS (quadrant IV), whereas 6.2% were in quadrant I. Of all simulations, 94.1% were under a WTP threshold of $10 737 per hospital rehospitalization avoided. If the WTP threshold was increased to the maximum of $17 830 as reported in Osenenko et al,^2^ a negligible amount of 0.5% were additionally below the threshold. The standard deviation stabilizes at 500 iterations and increasing the number of iterations did not change the result. Over the 1000 iterations, the median cost of care was $160 014 (95% CrI: $112 733; $223 887) for HFMS and $167 450 (95% CrI: $121 381; 230 938) for SOC. The median number of hospital readmissions was 1.072 (95% CrI: 0.697; 1.628) for HFMS and 1.197 (95% CrI: 0.838; 1.708) for SOC.

The incremental effect was measured as hospitalization avoided where a negative difference in number of hospitalizations indicates positive effectiveness. The effect of HFMS was implemented for the first 90 days of the model, the results from this time frame were a cost-saving of $949 ($14 333 SOC vs $13 384 HFMS) and 0.145 rehospitalizations avoided (0.386 SOC vs 0.241 HFMS).

**Figure 3.**
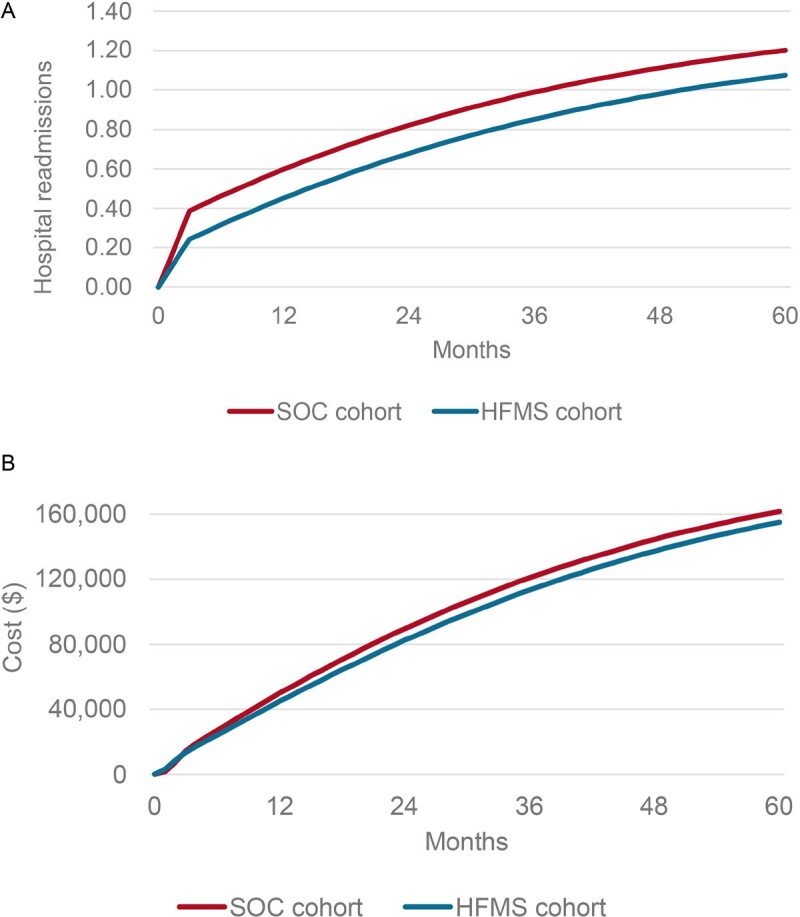
Cumulative Results of the Model per Patient for SOC and HFMS Arms for Hospital Readmissions (**A**) and Cost per Patient (**B**) Abbreviations: HFMS, Heart Failure Management System; SOC, standard of care.

When examining the costs by month in the simulation, we see that costs are initially higher in the HFMS arm until circa the second month. After this time, total costs are lower with HFMS (**Figure 3**). The initial higher costs likely reflect the cost of the HFMS device. Findings indicate that HFMS is cost-saving from the first year. The hospital readmission prevalence demonstrated a consistently lower value for the HFMS population (**Figure 3**), but this was driven by the initial reduction in hospitalization at the start of the model.

**Figure 4.**
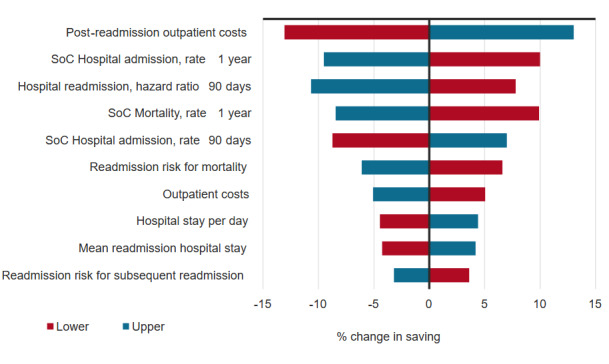
Results of the One-Way Sensitivity Analysis Abbreviations: LOS, length of stay; PSA, probabilistic sensitivity analysis; SOC, standard of care.

The one-way sensitivity analysis found that the model input with the largest impact on the results would be the outpatient care costs after readmission (**Figure 4**). A 10-percentage-point change in this input resulted in a change in the deterministic ICER of around 13%. This was followed by the hospital readmission rate at 1 year and the 90-day hazard ratio for readmissions for HFMS vs SOC.

## DISCUSSION

Our analysis found that HFMS is cost-effective compared with the current SOC for at-home monitoring of patients with heart failure in the United States. In this model, the use of HFMS reduced readmissions as well as the total cost of care over 5 years for patients recently diagnosed with heart failure. In the base case and in 93.4% of model iterations, HFMS dominated SOC monitoring.

Outcomes of this model align with findings from other studies on monitoring devices for heart failure.^16,23,24^ Not all remote monitoring interventions, however, have comparable effectiveness. Bui and Fonarow highlighted this when they identified that, although meta-analyses have shown a benefit for telemonitoring in ambulatory heart failure patients, randomized controlled trials of a telephone-based interactive voice-response system did not lead to significant changes in patient outcomes.^7^ The difference here, one may surmise, lies in the requirement for active patient involvement in reporting their data. Such a telemonitoring concept is fallible to low patient compliance, whereas automated, remote monitoring of objective parameters is not.

Hospital readmission is a critical factor in understanding the burden of heart failure. It is well known that the hospital readmission and subsequent inpatient costs substantially contribute to the financial burden of heart failure.^3^ The one-way sensitivity analysis highlighted the influence of hospital readmissions on the results, as they were among the three top factors. Hospital readmission also serves as a proxy for the effectiveness of the treatment that the patients are receiving. Although many readmissions are not avoidable, preventing avoidable hospital readmissions is an important part of reducing heart failure burden. The BMAD trial reported a reduction in hospital readmissions with HFMS.^14^ Due to impact of HFMS on readmissions, it is logical that changes in this input would impact the ICER. This is supported by the one-way sensitivity analysis results, as the model is sensitive to changes in the hospitalization hazard ratio.

Reducing hospital readmissions is part of the value of monitoring both for the payer and for the patients. Some patients that would be readmitted could perhaps be redirected to the physician’s office with the help of remote monitoring and management. Monitoring may also give information rapidly enough that deteriorating health can be caught earlier.^11^ It is important to note that remote management does not replace hospital readmissions entirely, as some patients will still need to be readmitted and moved to more invasive treatment.

Remote management also has indirect benefits, as highlighted in a systematic literature review on remote monitoring in chronic diseases.^25^ The patients felt more at ease and secure. Remote monitoring also empowered patients to improve their self-management and to be more involved in shared decision making.^25^ However, it was mentioned that the device’s ease of use and trust in the device are important to realize these benefits.

Heart Failure Management System is a new device, and this is the first analysis of the cost effectiveness of this device in any setting. The BMAD trial illustrated a greater increase in quality of life for patients in the active intervention arm.^10^ The measurement of thoracic fluid content and the electrocardiogram could assist physicians in making more informed decisions about treatment plan changes. Barriers to HFMS implementation may be similar to those of other medical devices, such as the integration with the healthcare system, and clinician buy-in.^26^ This analysis took a US-specific perspective. The US setting is in many ways unique, and these results may not be generalizable to other countries.

Furthermore, cost-effectiveness analyses for wearable monitoring devices in heart failure are sparse, providing little literature against which to compare. Due to the scarcity of some data, certain assumptions (as detailed in the Methods) had to be made during model development. The rates used after 90 days remained the same and were used for the rest of the time horizon, which may underestimate the benefits of the device and not accurately capture the changes in the rates over time.

Although this model was built to good practice guidelines, it cannot attempt to completely nor accurately reflect the patient care pathway and its outcomes that are realized in real life. Every model, however, is a good approximation of real life and should be treated as such. This model focuses on a collective cohort, and therefore individual patients and their unique path is not considered or tracked. Outcomes from the base case reflect one, potentially realistic scenario. Potential biases within the base case may come from the source data. There is currently one trial on HFMS, which enrolled 522 patients.^14^ It may be that these patients do not reflect the entirety of the population with heart failure in the United States. The study also compares time to first hospitalization and not overall hospitalizations. The model addressed this by separating first hospital readmission from subsequent ones. Furthermore, the cost data was drawn from different sources and there may be heterogeneity in reporting.

The sensitivity analyses were conducted to assess the influence of these assumptions on the results. During the sensitivity analyses, it was found that 0.4% of the results were not cost-effective, which is partly due to some uncertainty in the model inputs after 90 days. Additional data for longer time frames would be needed to check the trends demonstrated here.

Further research is needed to determine whether these predicted downstream benefits can be realized. This should include the analysis of more long-term outcomes in this population. This would allow us to better inform the model on whether there are differences in event rates with HFMS and SOC between 90 days and 1 year (or potentially beyond). Real-world data on the use of monitoring devices would also be beneficial as performance in clinical studies might not fully represent clinical use. For example, the exclusion and inclusion criteria tend to increase internal validity at the expense of generalizability (external validity).^27^

## CONCLUSION

By preventing unnecessary hospital readmissions, HFMS is expected to reduce costs and improve effectiveness over the SOC, supporting the use of HFMS for outpatient care of heart failure patients.

### Disclosures

A.B.S. and U.S. are employees, and R.S. is the CEO, of Coreva Scientific GmbH & Co KG, which received consultancy fees for performing, analyzing, and communicating the work presented here. A.V. is an employee of ZOLL Medical Corporation. P.J.M. is a consultant to ZOLL Medical Corporation. D.B. has been the recipient of ZOLL research grants and speaker fees. B.P. reports that they have no relationships relevant to the contents of this paper to disclose.
